# Early biting and insecticide resistance in the malaria vector Anopheles might compromise the effectiveness of vector control intervention in Southwestern Uganda

**DOI:** 10.1186/s12936-015-0653-z

**Published:** 2015-04-09

**Authors:** Patrick Ojuka, Yap Boum, Lise Denoeud-Ndam, Carolyn Nabasumba, Yolanda Muller, Michael Okia, Juliet Mwanga-Amumpaire, Pierre De Beaudrap, Natacha Protopopoff, Jean-François Etard

**Affiliations:** Epicentre Mbarara Research Centre, Mbarara, Uganda; Epicentre, Paris, France; National Malaria Control Programme Ministry of Health, Kampala, Uganda; Mbarara University of Science and Technology, Mbarara, Uganda; TransVIHMI IRD UMI 233-INSERM U 1175-Montpellier University, Montpellier, France; Department of Disease Control, London School of Tropical Medicine and Hygiene, Keppel Street, London, UK

**Keywords:** Malaria, Plasmodium falciparum, Anopheles, Biting, Uganda, Insecticide resistance

## Abstract

**Background:**

Southwestern Uganda has high malaria heterogeneity despite moderate vector control and other interventions. Moreover, the early biting transmission and increased resistance to insecticides might compromise strategies relying on vector control. Consequently, monitoring of vector behaviour and insecticide efficacy is needed to assess the effectiveness of strategies aiming at malaria control. This eventually led to an entomological survey in two villages with high malaria prevalence in this region.

**Methods:**

During rainy, 2011 and dry season 2012, mosquitoes were collected in Engari and Kigorogoro, Kazo subcounty, using human landing collection, morning indoor resting collection, pyrethrum spray collection and larval collection. Circumsporozoite protein of *Plasmodium falciparum* sporozoites in female *Anopheles* mosquitoes was detected using ELISA assay. Bioassays to monitor *Anopheles* resistance to insecticides were performed.

**Results:**

Of the 1,021 female *Anopheles* species captured, 62% (632) were *Anopheles funestus* and 36% (371) were *Anopheles gambiae s.l*. The most common species were *Anopheles gambiae s.l.* in Engari (75%) and *A. funestus* in Kigorogoro (83%). Overall, *P. falciparum* prevalence was 2.9% by ELISA. The daily entomological inoculation rates were estimated at 0.17 and 0.58 infected bites/person/night during rainy and dry season respectively in Engari, and 0.81 infected bites/person/night in Kigorogoro during dry season. In both areas and seasons, an unusually early evening biting peak was observed between 6 - 8 p.m. In Engari, insecticide bioassays showed 85%, 34% and 12% resistance to DDT during the rainy season, dry season and to deltamethrin during the dry season, respectively. In Kigorogoro, 13% resistance to DDT and to deltamethrin was recorded. There was no resistance observed to bendiocarb and pirimiphos methyl.

**Conclusions:**

The heterogeneity of mosquito distribution, entomological indicators and resistance to insecticides in villages with high malaria prevalence highlight the need for a long-term vector control programme and monitoring of insecticide resistance in Uganda. The early evening biting habits of *Anopheles* combined with resistance to DDT and deltamethrin observed in this study suggest that use of impregnated bed nets alone is insufficient as a malaria control strategy, urging the need for additional interventions in this area of high transmission.

## Background

Uganda is one of the most affected country with Malaria, 90% of the population resides in endemic areas (WHO malaria report 2014). Southwest Uganda is classified as very high transmission setting [1,2]. A decrease in malaria prevalence has been observed in southwestern Uganda [3], as described in many countries of sub-Saharan Africa [4]. This decline has been attributed to use of long-lasting insecticide nets (LLINs), indoor residual spraying (IRS), the introduction of artemisin-based combination therapy (ACT) for malaria treatment and intermittent preventive treatment (IPTp) during pregnancy. There is however a large spatial heterogeneity in transmission and some rural areas have reported malaria prevalence higher than 70% in children under five [3]. In Tororo, Eastern Uganda, despite 98% LLIN usage reported, malaria incidences were still high, with a median incidence of 5.3 per person-year [5]. In addition to ITN coverage and IRS to reduce the vector density, host-seeking malaria vectors are still found indoors in high numbers due to the house design that favour mosquito entry [6].

Change in biting behavior has been observed following implementation of vector control intervention in Benin [7]. Early evening or morning biting when the population is not protected by LLIN could sustain residual malaria in area with high coverage of net [8,9]. It has also been reported in Kenya and Tanzania that vector control intervention might be less effective against *Anopheles arabiensis,* another major malaria vector that is less killed by LLIN and ITN treated with pyrethroids as compared to their counterparts *Anopheles gambiae* and *Anopheles funestus* [10,11].

This might explain why malaria prevalence or incidence remained high even in areas where LLIN coverage is high [7]. There is an urgent need to assess the mosquito behavioural dynamics in order to come up with an appropriate control strategy to the malaria transmission. However, there is limited data on mosquito behaviours that could guide the implementation of adapted strategy to control malaria in Southwestern Uganda.

In the past decade, with the scale up of vector control intervention, emergence of insecticide resistance especially pyrethroid, has been observed in Uganda [12] and all over Africa. Some studies have shown reduced efficacy of LLINs against resistant vector populations [13,14]. In Uganda, a study conducted during 10 years show a continuous drop in mortality of *An. gambiae* exposed to pyrethroid impregnated nets under laboratory conditions [15]. Control failure attributed to insecticide resistance has been observed in IRS programmes in South Africa [16,17] and in the island of Bioko [18]. More studies are required to determine the impact of pyrethroid resistance on the effectiveness of LLINs in Uganda.

The results of a cross sectional survey carried out in the greater Mbarara in 2010 showed high malaria prevalence levels in Kazo subcounty in Kiruhura district despite reported bednet usage [3]. The present study aimed to assess vector behaviour and insecticide resistance status of the main vectors that could explain the high malaria prevalence in the area.

## Methods

### Study area

**Figure 1:**
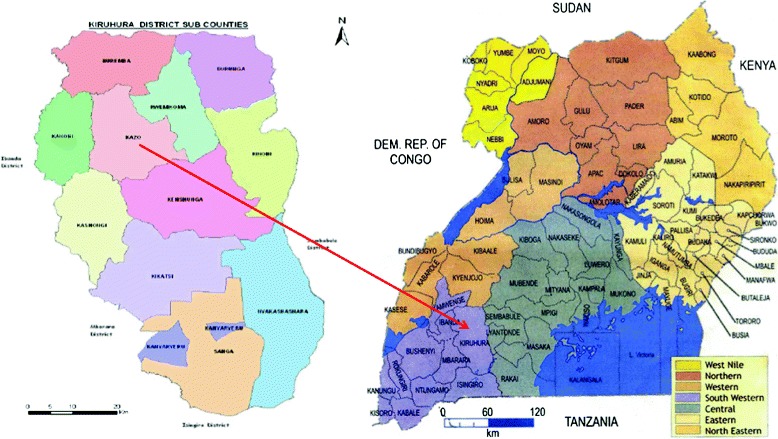
**Map of Kiruhura district showing Kazo subcounty, Uganda.**

The study was conducted in Engari-Kakindo (0° 1′ 57.44′ S, 30° 40′ 33.74′E; Altitude = 1255 m) and Kigorogoro (0° 2′ 9.85′ S, 30° 48′ 28.54′E; Altitude = 1271 m) villages in Kazo subcounty located in Kiruhura district of southwestern Uganda (Figure 1). The area has high and perennial malaria transmission, with two peaks following the rainy seasons.

Malaria prevalence decrease from 43% in 2004 to 23% in 2010 reported in children less than five years of age. There was however high heterogeneity with nearly 10% of the area having prevalence greater than 50% [3].

The district lies within the great East African geographical rift valley. The estimated population of 290,400 (2011) and 300,800 (2012) inhabitants is mainly rural [19]. Their main occupations are agriculture, cattle rearing and cropping. The climate is equatorial with a long bi-modal rainy season from mid-August to December and from March to May. Rainfalls average 1,200 mm per annum and the temperatures 25-27°C. The households were mainly grass thatched, “igloos”, semi-permanent and permanent houses. Each household has its own water facility that serves both animal and human consumption in addition to communal valley dams and troughs. The larval habitats in the two sites were different. Engari was seen to have short shrub like vegetation with usually open water pools, foot and hoof prints. Kigorogoro however exhibited papyrus type of vegetation, swamps and large water pools usually with an emergent vegetation or shaded environment. This could explain the Anopheles species variation within the two sites. A survey carried out in Nyabushozi County, Kiruhura district, with similar environmental dynamics to Kazo found Anopheles species variations in two different villages. *Anopheles gambiae* was dominant species in Mugore village and *An. funestus* dominated in Kakyeera village [13]. The main malaria prevention methods used in these areas include use of treated and non-insecticide treated bed nets. The bed net usage in children under 5 years old was 44.5% in 2010 [3]. No indoor house spraying (IRS) with pyrethroids has ever been conducted in Engari and Kigorogoro.

### Entomological methods

#### Selection of the houses and seasons

Two rounds of mosquito captures were done in Engari in April 2011 during the rainy season and in February 2012 during dry season and one round in Kigorogoro during the dry season. Monthly rainfall data obtained from the Mbarara meteorological station showed 132.1 mm of rainfall within 16 rain days during April 2011 and 23.3 mm of rainfall within 5 rain days during February 2011. The maximum and minimum temperatures reported from the same source were 26.5°C and 16.0°C during April 2011 and 30.8°C and 15.7°C during February 2011 respectively (unpublished data). The February 2011 meteorology data was used to predict the February 2012 weather.

Twenty houses in each village were selected following specific criteria which includes; older house constructions, outside commercial centres, close to lowland and without kitchen inside the main houses to avoid the effect of smoke on mosquito collection. Mosquito collections were performed during a preliminary study in Engari during the rainy season (April 2011).

#### Mosquito collection and identification

Human landing catches (HLC) were carried out in three houses per village during six days in the rainy season in Engari and ten days during the dry season in Engari and Kigorogoro. Indoor HLC was performed between 6 p.m.- 6 a.m. and outdoor collection between 6 p.m. to midnight.

Morning indoor resting catches (MIRC) was performed in six houses in Engari and Kigorogoro, different from the ones of HLC, for ten days during dry season and six days during the rainy season in Engari. The collections were done using a hand held aspirator between 6-8:30 a.m. In addition Pyrethrum spray catches (PSC) were carried out in 20 houses in each village on the last day of mosquito collections between 8:30 a.m. and midday during the two seasons.

Female adult *Anopheles* mosquitoes used for WHO resistance testing were reared from larvae, as described in Service et al [14].

Morphological keys were used to identify malaria vectors collected [15,20]. All mosquitoes belonging to the *Anopheles* family were kept in individual tubes containing silica gel for further laboratory testing. Only females heads and thoraxes from HLC, MIRC and PSC were processed for detection of circumsporozoite protein (CSP) of *P. falciparum* sporozoites using an enzyme-linked immunosorbent assay (ELISA) technique [16]. According to Echodu *et al*., 14.2% (n = 1544) of *An. gambiae s.l.* analysed using PCR were identified as *An. gambiae S.S.* and no *An. arabiensis* was found. In addition, 20% (n = 270) of *An. funestus* group analysed were found to be *A. funestus*, *An. leesoni*, and *An. parensis* using PCR in Nyabushozi County, Kiruhura district [13]. These species variations observed in a similar setting to this current study gives a comparison picture of what would have been expected in Kazo subcounty, Kiruhura district.

#### Entomological indicators

Human biting rates (HBRs) for *Anopheles* were calculated as number of bites per human per night obtained from HLC. The calculation of outdoor HBR considered that an average villager enters the house by 10 p.m. latest. Sporozoite rates (SRs) were the proportions of female *Anopheles* found positive to CSP antigens. Daily entomological inoculation rates (EIRs i.e. number of infected bites per human per night) were obtained using the average number of *Anopheles* CSP positive collected by HLC per nights of collection for each village and season. Monthly EIRs were extrapolated for April 2011 and February 2012. The indoor resting density (IRD) at the house level for each *Anopheles* species was calculated as the number of *Anopheles* females captured at each round of MIRC divided by the total number of houses inspected, in each village and season.

#### Insecticide bioassays

The insecticide resistance status of the malaria vectors was assessed using mosquitoes collected by larval collection and adults from HLC and/or MIRC when low numbers of larvae were found.

Insecticide susceptibility assays were performed using two to three days old adult female *A. gambiae s.l* and *A. funestus* fed on 5% glucose and adults of unknown ages from HLC and MIRC. Five replicates of 20 adult female *Anopheles* were exposed to each of the four different insecticide-impregnated papers (DDT 4%, bendiocarb 0.1%, deltamethrin 0.05% and pirimiphos methyl 1.0%) in standard WHO test tubes. One batch of 20 adult female *Anopheles* was subjected to untreated papers to act as controls. All tests were undertaken at 25°C ± 2°C and 70–80% relative humidity during one hour.

Cumulative knockdown (KD) counts were recorded every 5-10 minutes for the respective exposure periods. After exposure, mosquitoes were supplied with 5% glucose solution, and mortality was recorded at 24 hours post-exposure. KT50 was the time after which 50% of the *Anopheles* tested were knocked down and KD60 as the proportion of *Anopheles* knocked down after 60 minutes [17,18].

### Data analysis

Data were double entered using Epidata V.3.1 (Epidata, Odense, Denmark). Statistical analysis was performed using Stata 12 (College Station, Texas). Descriptive statistics were presented for each village and season of capture. Fisher exact test was used to compare proportions.

### Ethical considerations

The study obtained ethical clearance from the Mbarara University, Faculty of Medicine research committee (FREC), Mbarara institutional review committee and Uganda national council of science and technology (UNCST). Informed written consent from the heads of the households and from the collectors were obtained. In addition malaria prophylaxis (doxycycline) was provided to the collectors performing HLC. Blood smear slides and rapid diagnostic tests each week from the collectors for malaria diagnosis. If the blood slides were found positive, the participant was treated with artemether-lumefantrine (Coartem®) according to the Ugandan national guidelines. A follow-up blood slide smear check was performed on a weekly basis until day 42 when the RDT turned out negative to ensure that all parasites were fully cleared; if not, the worker was referred to the hospital to receive the second line treatment.

## Results

### Vector densities and transmission

Overall 1,021 female *Anopheles* species were collected during the two rounds. Of these, 62% (n = 632) were members of the *A. funestus* group, 36% (n = 371) specimens belonged to the *A. gambiae s.l.* and the remaining 2% belonged to other *Anopheles* subspecies (Table 1). Forty-nine (49%) of the total female *Anopheles* was collected using HLC, 37% with MIRC and 14% with PSC.

**Table 1:** **Anopheles species collected in each village and season**

	**Total N = 1021% (n)**	**Engari, rainy season N = 133% (n)**	**Engari, dry season N = 236% (n)**	**Kigorogoro, dry season N = 652% (n)**	P*
Species					<0.0001
A. *gambiae*	36% (371)	87% (116)	67% (159)	15% (96)	
*A. funestus*		11% (15)	31% (72)	83% (545)	
Other Anopheles	62% (632) 2% (18)	2% (2)	2% (5)	2% (11)	

*P was calculated with the Fisher exact test.

During dry season in Engari, 67% of collected female Anopheles were *A. gambiae s.l*., 31% were *A. funestus* and 2% were other *Anopheles* species. In Kigorogoro site, 83% of the female Anopheles collected were *A. funestus* and 15% were *A. gambiae s.l*. The species were differently distributed between villages (P < 10^-4^, Fisher exact test). Furthermore, there was a statistically significant difference in the distribution of *A. gambiae* and *A. funestus* between the dry season and rainy season in Engari site (P < 10^-4^, Fisher exact test).

The indoor resting density (IRD) for *A. gambiae s.l* and *A. funestus* per house during rainy season in Engari and dry season in Engari and Kigorogoro are summarized in Table 2. ELISA testing for CSP antigen was performed in 975 out of the 1003 (96%) of the *Anopheles* captured. Overall, sporozoite rate was 2.9% (95% CI, 1.9%-4.1%), (Table 2). During the rainy season in Engari, only *A. funestus* was found positive for *P. falciparum* CSP. During dry season, SR was 4.8% and 2.7% in Engari and Kigorogoro. There was no statistically significant difference in SR between species (P = 0.693, Fisher exact test), nor between villages and seasons (P = 0.11).

**Table 2:** **Vector densities and transmission in each village and season**

	**Engari, rainy season N = 133**		**Engari, dry season N = 236**		**Kigorogoro, dry season N = 652**	
	***A. gambiae s.l***	***A. funestus***	***A. gambiae s.l.***	***A. funestus***	***A. gambiae s.l.***	***A. funestus***
Indoor Human biting Rate, per person per night (HLC)	8.08	1.08	5.93	2.53	2.38	24.33
Outdoor Human Biting Rate, per person per night (HLC)	0.13	0.04	0.33	0.1	0.15	0.73
Indoor resting density, per day per house (MIRC& PSC)	1.53	0.26	1.06	0.53	0.8	3.14
Sporozoite rate,% (HLC, MIRC and PSC)	0%	0.8%	6.3%	1.4%	2.2%	2.8%
Daily Entomological Inoculation Rate, per person per night (HLC)	0	0.17	0.58	0	0.03	0.78
Monthly Entomological Inoculation Rate, per person per month (HLC)	0	5.1	16.82	0	0.87	22.62

Note: Methods use for *Anopheles* collection: HLC, human landing collection. MIRC, morning indoor resting collections. PSC, pyrethrum spray collection.

**Figure 2:**
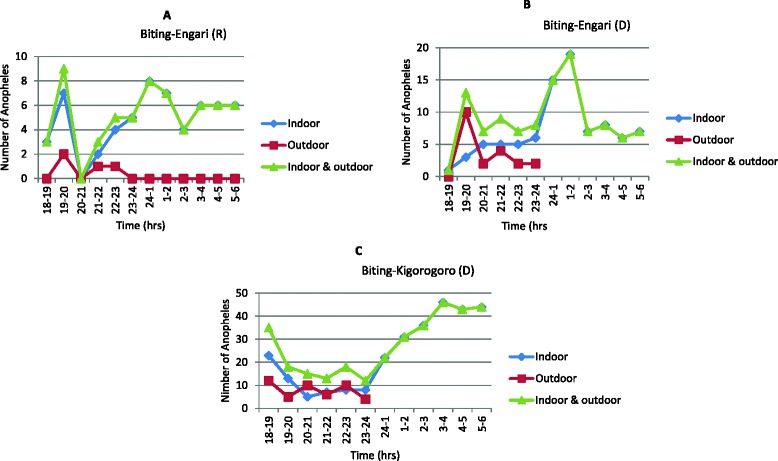
**Biting peaks and resting preferences of** ***Anopheles*** **. A** Biting peaks of *Anopheles* mosquitoes in Engari during rainy season; **B** Biting peaks of *Anopheles* mosquitoes in Engari during dry season; **C** Biting peaks of *Anopheles* mosquitoes in Kigorogoro during dry season.

Figure 2 shows the HBRs calculated from HLC for the different species and types of collection (indoors and outdoors), for each village and season. The minimum was observed in Engari during the dry season (8.9 bites per human per night, mostly caused by *A. gambiae*), while the maximum was observed in Kigorogoro during the dry season (27.6 bites per human per night, mostly caused by *A. funestus*).

The EIRs obtained from HLC were respectively 0.17, 0.58 and 0.81 bites per person per night during the rainy, dry seasons in Engari and dry season in Kigorogoro. No transmission was observed outdoors except during dry season in Kigorogoro (0.06 infected bites per night). The corresponding monthly EIRs were extrapolated at 5.1 bites per person per month in April 2011 (rainy season) in Engari, and at 16.82 and 23.5 bites per person per month in February 2012 (dry season) in Engari and Kigorogoro respectively (Table 2).

### Biting behaviour and resting preference

In Engari, the biting peak of *Anopheles* mosquitoes was observed between 7-8 p.m. and midnight to 1 a.m. during rainy season, (Figure 2A). During dry season, the early evening peak was similar to that of rainy season with a slight shift during late hours; 1-2 a.m. (Figure 2B). In Kigorogoro, an early evening biting peak between 6–7 p.m. and early morning peak between 3-4 a.m. during dry season was observed (Figure 2C). One circumsporozoite protein positive Anopheles was found during the 7-8 p.m. outdoor collection in Kigorogoro; indicating early malaria transmission. *A. gambiae s.l* and *A. funestus* preferred to bite and rest indoors more than outdoors (Figure 2A,B,C).

### Resistance to insecticides

Eight hundred forty (840) female *Anopheles* mosquitoes, mainly *A. gambiae* complex in Engari and *A. funestus* group in Kigorogoro sites were exposed to DDT, bendiocarb, pirimiphos methyl and deltamethrin, WHO insecticide impregnated papers. The results from DDT showed a 15% mortality rate after 24 hours during rainy season in Engari site indicating resistance.

During dry season, results from DDT and deltamethrin tests showed a 66% and 88% mortality respectively in Engari. In Kigorogoro, results from DDT and deltamethrin tests both showed 87%mortality, whereas 100% mortality was observed with Bendiocarb and Pirimiphos Methyl, suggesting full susceptibility.

## Discussion

*Anopheles gambiae s.l.* and *A. funestus* were found to be the principal vectors in Engari and Kigorogoro, which are the common species reported in Africa [21]. This is in line with a survey finding from Echodu et al in Nyabushozi County in Kiruhura district, whose environmental setting is similar to Kazo subcounty; *A. gambiae* complex (n = 1544) and *A. funestus* group (n = 186) were the most prevalent vectors [13].

The dominant malaria vector species were different in the two sites during dry season, with *A. gambiae s.l.* prevailing in Engari and *A. funestus* prevailing in Kigorogoro. This could be attributed to the difference in the breeding sites. Engari had open water pools, foot and hoof prints with short shrub like vegetation. Kigorogoro on the other hand had papyrus type of vegetation, swamps and large water pools with emergent vegetation or shaded. Elsewhere, *A. funestus* has been found to be closely associated with aquatic vegetation [22] while *A. gambiae s.s.* and *A. arabiensis* inhabit small and sunlit temporary water pools [20,22]. Proportion of *A. gambiae* was even higher during the rainy season in Engari. Unfortunately, results for rainy season could not be compared between the two sites as mosquito collections were not performed in Kigorogoro during rainy season. Molecular species identification was not performed for the *A. gambiae* complex, therefore it is not possible to know if the proportion *A. gambiae*/*A. arabiensis* was different between the season and the site and could also explain the variation in phenotypic resistance observed in the WHO test.

Mosquitoes expectedly preferred to bite and rest indoors than outdoors. During the rainy season, the biting behaviours of *Anopheles* showed an early peak between 7-8 p.m., (indoor and outdoor), a time where the population is not protected by LLIN. The late peak between midnight to 1 a.m. can worsen the situation especially in areas where there are no records of LLIN mass distribution to the vulnerable group. Furthermore, a similar situation was observed during dry season; early and late biting peaks occurred between 7-8 p.m. and 1-2 a.m. respectively in Engari. However, the early evening biting peak in Kigorogoro site was earlier than that of Engari during same dry season where an early peak occurred between 6-7 p.m. and late peak between 3-4 a.m. Indeed the early biting observed is more worrying since transmission occurs when the population is unprotected from the bites. Of note, studies done in the Equatorial Guinea reported a late evening biting peak between 9 -10 p.m., both indoor and outdoor [23]. Moreover, recent studies done in Benin provide evidence for a substantial diurnal host-biting behaviour of a major malaria vector in Africa. According to the authors, a 26% proportion of *A. funestus* collected after 6 a.m. was suggestive of diurnal biting activity [7]. These figures showing a shift from nocturnal to diurnal biting behaviour of mosquitoes poses another threat to the malaria control strategies. In the present study human landing collection were stopped at 6 a.m. and therefore it was not possible to show possible diurnal biting activity while from the data it seems that mosquitoes were still active at 6 a.m. Overall, studies carried out elsewhere on biting behaviours in Uganda present results quite different to this present survey and they could be attributed to environmental factors. A study on biting pattern and seasonality of *A. gambiae s.l.* and *A. funestus* mosquitoes in Kamuli district, located in the eastern Uganda showed a biting peak between 11 p.m. and 5 a.m. [24] which is quite different from that found in Kazo subcounty, Kiruhura district, Uganda.

Monthly EIR (mEIR) was higher during the dry season than the rainy season with mEIR over 16 infectious bites per month per person. A study carried in the eastern districts of Uganda, Jinja and Tororo and southwestern district, Kanungu showed monthly *pf*EIR with strong seasonal signals occurring between May-June and October-December, usually at the end of the rainy season. During the dry season, the transmission was mainly due to *A. gambiae* in Engari and to *A. funestus* in Kigorogoro. Previous studies have indicated that *A. gambiae s.l*. biting activity is compatible with malaria transmission occurring only at the end of the rainy season [25,26]. Moreover, there is also evidence that *A. funestus* play an important role in malaria transmission during the dry hot season [20,27,28]. The present study is in agreement with the above reports since it found *A. funestus* responsible for both indoor and outdoor early malaria transmission between 7-8 p.m. when the population is unprotected by LLIN. Complex interactions of many factors have been attributed to influence the abundance of mosquitoes and the EIR, including temperature, altitude, rainfall, urbanization and the availability of suitable larval habitats [29-31]. As a result, EIRs are subjected to strong seasonal variations [32]. The overview of a survey carried out in seven sentinel sites of Uganda, showed monthly EIR ranged between 0.0 infected bites per person per night (ib/p/n) in Kanungu, southwestern Uganda to 9.7 (ib/p/n) in Apac district, northern Uganda [1], which is in line with this current study.

An important issue is the level of resistance to DDT and deltamethrin in both study areas. Fifteen percent mortality was observed in *A. gambiae* complex against DDT during the rainy season in Engari while the mortality was higher (over 60%) during the dry season in the same area. In Kigorogoro, over 80% mortality mainly from *A. funestus* group was observed which still poses a threat since these insecticides are the ones currently used in indoor residual spraying and impregnation of bed nets respectively. However, recent findings in Benin [8] showed *A. funestus* population fully susceptible to deltamethrin, the insecticide used in Permanet 2.0 bed net. According to the author, 100% KD after 60 minutes was suggestive of absence of any KD resistance alleles. In this present study, mosquitoes were fully susceptible to Bendiocarb and Pirimiphos methyl with 100% mortality after 24 hours monitoring prior to exposition to the insecticides. The resistance status could be attributed to the fact that kazo communities are cattle keepers and spray their cattle from time to time with acaricides to eliminate ticks, mites and other vermin. This argument could be true especially if the *Anopheles* sibling species were identified as *A. arabiensis* that is usually related to animal presence. As described by Echodu *et al.* [13], farmers apply synthetic pyrethroids, such as deltamethrin, on their cattle for control of both ticks and tsetse flies. This may suggest that the continuous use of these acaricides near households might contribute to the resistance of mosquitoes to the insecticides since their chemical composition and class maybe the same. Therefore, rotation of insecticides/pesticides would reduce resistance spread in the area.

## Conclusions

The principle malaria vectors identified in this study were *A. gambiae s.l.* in Engari and *A. funestus* in Kigorogoro, with an overall 2.9% *P. falciparum* prevalence and daily EIRs ranging from 0.17 to 0.81 infective bites per person per night. In both areas, an unusually early biting peak was observed between 6-8 p.m. with outdoor transmission between 7-8 p.m. and resistance to DDT and deltamethrin was documented. Taken together, these results suggest that relying on bed net use alone as a malaria control strategy may not offer sufficient protection urging the need for additional interventions like indoor residual spraying, insecticide rotation and human behavioural change in this area of high transmission.
